# Supplementary figures and images for: Differential lncRNA/mRNA expression profiling and ceRNA network analyses in amniotic fluid from foetuses with ventricular septal defects
Source: PeerJ. 2023 Feb 27;11:e14962. doi: 10.7717/peerj.14962 (PMC9979828; doi:10.7717/peerj.14962)

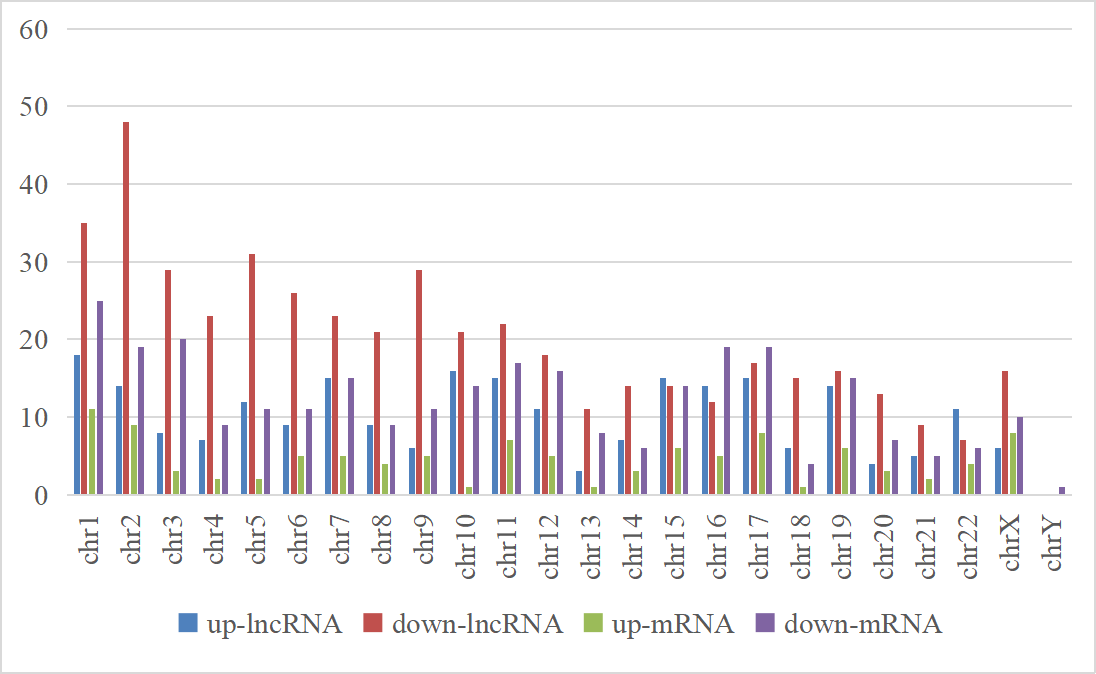

Supplement: Supplemental Information 7 — The abscissa represents different chromosomes, the ordinate represents number of differentially expressed genes, the color represents gene types. [file peerj-11-14962-s007.png]
